# Caries Experience in Primary and Permanent Dentition in Children Up to 15 Years of Age from Bosnia and Herzegovina—A Retrospective Study

**DOI:** 10.3390/children10040754

**Published:** 2023-04-21

**Authors:** Marija Obradović, Olivera Dolić, Vladan Milovanović, Nataša Karaman, Maja Mišić, Vesna Miljević, Sanja Matošević-Jajčanin, Slava Sukara, Predrag Kaurin, Nataša Knežević, Mirela Regoda-Šeranić, Darija Mijatović, Božana Galić-Pejić

**Affiliations:** 1 Department of Pediatric and Preventive Dentistry, Medical Faculty, University of Banja Luka, 78 000 Banja Luka, Bosnia and Herzegovina; 2 Public Health Center Kneževo, 78 230 Kneževo, Bosnia and Herzegovina; 3 Public Health Center Čelinac, 78 240 Čelinac, Bosnia and Herzegovina; 4 Public Health Center Doboj, 74 000 Doboj, Bosnia and Herzegovina; 5 Public Health Center Banja Luka, 78 000 Banja Luka, Bosnia and Herzegovina; 6 Department of Restorative Dentistry and Endodontics, Medical Faculty, University of Banja Luka, 78 000 Banja Luka, Bosnia and Herzegovina; 7 Institute of Dentistry, 78 000 Banja Luka, Bosnia and Herzegovina

**Keywords:** prevalence, mean dmft/DMFT, significant caries index-SiC index, male, female, children

## Abstract

The purpose of the study was to analyze caries experience in primary and permanent dentition in children up to 15 years of age located in Banja Luka, Bosnia and Herzegovina. Methods: The research was conducted as a retrospective cross-sectional study. Analyzes and comparisons of caries indices were performed using groups formed according to their gender (male—M and female—F) and age, i.e., the first group-children in early childhood, ≤5 years; the second group, middle childhood 6–8 years; the third group, preadolescents 9–11 years old; fourth group, adolescents 12–15 years old. Results: Overall prevalence of caries in primary dentition was 89.1%, while in permanent dentition, it was 60.7%. The overall mean decayed, missing, and filled teeth— dmft in male participants was 5.4, while in female participants, it was 5.1. By contrast, a higher overall mean DMFT was established in the female participants (2.7 vs. 3.0). Conclusions: We can see a high prevalence in all of the examined groups. In primary dentition, males examined during the course of the study had a higher overall mean dmft and the mean number of untreated decayed primary teeth, whereas females up to age 15 examined during the course of the study had more DMF teeth.

## 1. Introduction

Dental caries is a multifactorial disease with well-known primary and secondary etiological factors. Despite the established etiology related to this disease, more research is needed in order to ascertain the most high-risk groups and arrange for appropriate preventive strategies so as to prioritize the initial treatment and subsequent management of the condition and to optimize follow-up. Caries is also of great interest to researchers due to its global distribution. Significantly, oral diseases are present in about 3.5 billion people worldwide, while caries of permanent teeth occur in approximately 2.3 billion people, with approximately 530 million children suffering from caries of deciduous teeth [1].

The World Health Organization (WHO) recommends the implementation of periodic national studies on oral health that include the monitoring of ten parameters of oral health by precisely defined index age groups [2]. In accordance with WHO recommendations, the Public Health Institute of the Republika Srpska (RS), Bosnia and Herzegovina (B&H) issues an annual report entitled “Population Health Status”, in which it presents the diseases, conditions, and injuries identified by the dental healthcare services in the various regional centers located throughout the RS; however, the annual report issued by the RS presents this data without reference to the age or sex of the patients and lacks a detailed analysis of individual disease parameters [3]. Earlier research on the prevalence of caries in Bosnia and Herzegovina, including those conducted by the Department of Pediatric and Preventive Dentistry, Study Program in Dentistry at the Faculty of Medicine in Banja Luka (BL), shows that 64.7% of children under 6 years of age have caries on their deciduous teeth [4]. Research on 12-year-olds shows that the mean number of decayed, missing, and filled permanent teeth—mean DMFT ranges from 4.9–6.7, depending on whether they live in an urban or rural environment, with urban children having a lower DMFT [5]. Similar results have been identified by dental professionals in the other government entity of Bosnia and Herzegovina, the Federation, who have found average values of the mean DMFT/dmft in children aged 6 years (mean dmft: 6.7), 12 years (mean DMFT: 4.2), and 15 years (mean DMFT: 7.6) [6]. The latest published study showed the prevalence and severity of early childhood caries in preschool children in the Federation in a total of 165 preschool children aged 3 to 5 years. The mean dmft (decay, missing, filled teeth index) was 6.8, and the percentage of caries-free children was 17.0% [7].

Studies have shown a high prevalence of caries in children on the territory of Bosnia and Herzegovina as a whole. Due to such a prevalence and the fact that much of the published data is currently outdated, there is a need to analyze the current situation with respect to the oral health of children and adolescents.

Moreover, there is also a lack of research dealing with the role of a patient’s gender/sex in the prevalence of oral diseases in B&H. Globally, there have been studies related to gender/sex and carious and periodontal diseases [8,9,10,11,12,13,14,15,16,17]. The results of such studies tend to show considerable variability without a clear consensus to be found in the literature [13,14,15,16,17]. Notably, a recent analysis conducted by Elamine and associates showed that the female sex was associated with a higher risk in six studies, whereas males were at a higher risk in eight of the studies [17]. Therefore, additional sex/gender-based regional and national research is needed in order to define the “disease burden” at the individual level and to identify individuals who may be at higher risk based upon their gender.

The aim of our research was to analyze caries prevalence, mean dmft/DMFT and Significant caries index-SiC in Primary and Permanent dentition in children up to 15 years of age, patients of the Pediatric Dental Clinic (Dental Clinic), Faculty of Medicine, University of Banja Luka (BL), Republika Srpska (RS), Bosnia and Herzegovina (B&H).

## 2. Materials and Methods

We conducted our research using a retrospective cross-sectional study approach, which included examinees up to 15 years of age (the youngest being 2.8 years of age and the oldest being 14.11 years of age) of both sexes, with examinees identifying as either male or female. Prior to the research, written consent for conducting the research was obtained from the Ethics Committee (reference number 18/4.78/22) of the Faculty of Medicine, University of Banja Luka (BL).

The research sample was formed using data obtained from the Demographic Statistics, Statistical Bulletin (Bulletin), Republic Institute of Statistics, Republika Srpska, Bosnia and Herzegovina (B&H) [18]. According to the Bulletin, the average number of children (individuals aged 0–19 years of age) by sex and five-year age groups for 2018, 2019, and 2020 in the RS and B&H was N = 213,529. Using Cohron’s sample size estimate for the given population, we get the minimum sample size for research *n* = 384. Using the Kolmogorov–Smirnov test (KS = 0.233, *p* = 0.2) with a confidence interval of 95%, it can be established that there is no significant deviation of the population sample from the normal distribution. For the purpose of this research, information on the health status of the examinees’ teeth was obtained from the existing electronic health records (e-charts) of the Dental Clinics’ software, which were created as part of their regular clinical practice with respect to each patient during their initial visit to the clinic. For the purpose of our research, the data used was from e-charts collected in the period from January 2019 to January 2022. In our research, we only used information about the state of health of the examinees’ teeth without the use of any personal data or identifiers. All children up to the age of 15 who visited the Dental Clinic in the period from January 2019 to January 2022 participated in the research. The children come from different socio-economic backgrounds and different preschools and schools throughout the municipality of Banja Luka, and consequently, they formed a simple random sample.

Dental pediatric patients who visit the Dental Clinic of the Faculty of Medicine of Banja Luka are children with common oral health issues; children referred by orthodontists for dental health assessment or for the implementation of interceptive orthodontic procedures (such as serial extractions and approximal enamel reduction); children whose parents bring them for regular dental check-ups and for the application of caries preventive and prophylactic measures; and children having systematic dental examinations who regionally belong to our health clinic.

Five examiners (V.M., N.K., M.M., V.M., and S.M.J.) who performed baseline examinations have DMD (Doctor of Dental Medicine) diplomas (about 10–15 years of work experience) and, at that time, were on Pediatric Dentistry Specialty Training at the Dental Clinic. Their parent institutions are public school clinics where they perform systematic dental examinations of school children every year as part of their regular work. Prior to conducting the examinations, they were trained in using the Decayed Missing Filled Teeth/decayed missing filled teeth-DMFT/dmft index on 30 preschool and school children for the inter-consistency of the fieldwork investigators. During their Specialty Training at the Dental Clinic, they had lectures and assignments related to PowerPoint presentations on the assessment of dental health according to the World Health Organization-WHO criteria used in the design of this study. While performing examinations of the children, they were under the supervision of their mentors (M.O. and O.D.), who are associate professors having a combined 20 years of experience in using the Klein Palmer dmft/DMFT index for research [19]. Their mentors were previously trained, and calibrated on epidemiological surveys and their inter-examiner kappa value was >0.87. The five researchers (V.M., N.K., M.M., V.M., and S.M.J.) later collected data from the dental records, and all extracted data were double-checked by an associate professor (M.O.).

During a child’s first visit to a Dental Clinic, a detailed intraoral inspection was performed, and the condition of both the teeth and oral tissue was recorded in the appropriate “dentition status” on the e-chart. The examination of the teeth is conducted under the following standard conditions: it is completed on a dental chair, under artificial light, using a dental mirror, and by drying the teeth with air from an air/water dental syringe. The presence of caries is recorded in accordance with the guidelines set forth by the WHO [2], which involve impaired enamel integrity and the existence of a carious cavity. Clearly visible lesions with cavities on tooth surfaces were classified as dental caries (i.e., d3-level cavities), whereas changes in transparency, initial enamel demineralization with intact surfaces, and no cavitations were classified as intact teeth. The teeth were not professionally cleaned, although the children brushed their teeth before the examination. No radiographs were taken. A filling is considered to be a tooth that has been conservatively or endodontically treated from a previous disease without the presence or suspicion of secondary caries. Extraction is recorded if there has been a tooth extraction as a therapeutic measure due to the presence of caries or complications of caries. Extraction of a deciduous tooth as a result of physiological exfoliation is recorded as such and was not the subject of this research.

In the first phase, in which information was collected from the e-charts, our research involved a total of 774 children up to 15 years of age. As the minimum sample for testing was previously determined, two groups were formed in relation to primary and permanent dentition. Therefore, our criteria for inclusion of respondents in the group with primary dentition involved the presence of at least one primary tooth and an adequate level of cooperation that allowed for the completion of the examination in accordance with WHO criteria. In addition, the criterion for inclusion in the permanent dentition group was the presence of at least one permanent tooth and complete “dentition status”. The final sample for our analyses of primary teeth included a total of 530 children (the oldest female being aged 12.9 years of age and the oldest male being 12.8 years of age) and 493 children in the group for our analysis of permanent teeth.

After downloading all of the data concerning the existing “dentition status” from the e-charts and completing the database in Excel, we commenced further analysis using the following methodology.

Specifically, our analysis of the data was performed using the Klein-Palmer (Decayed Missing Filled Teeth/decayed missing filled teeth-DMFT/dmft) [19] system and the Significant caries index-SiC index according to Bratthall [20].

We performed our analyses and comparisons of indices upon groups according to sex and age (male—M and female—F), i.e., the first group was children in early childhood ≤ 5 years of age, the second group-middle childhood 6–8 years of age, the third group preadolescents 9–11 years of age, and the fourth group adolescents 12–15 years of age.

The results that we obtained regarding the caries index for each group were processed using statistical tests. The processing program used is SPSS 25.0. All data were processed using a confidence interval (CI) of 95% and a significance level of *p* < 0.05. A comparison of the mean dmft/DMFT and SiC index among different age groups was performed using One-Way ANOVA. The significance of the difference for the SiC index based upon the different groups identified by the respondent’s sex was statistically processed using the Student’s t-test for independent samples with sample validation and Tuckey correction. For the statistical processing of the mean DMFT/dmft index, the Z-test for independent samples was used. An assessment was made in the group of primary teeth and in the group of permanent teeth using the normalized absolute value of the proportional representation of the number of carious teeth in relation to the number of examined teeth for each type of tooth individually, with 95% CI.

## 3. Results

The distribution of the study subjects is presented in Table 1. The Primary teeth sample included 243 (45.9%) female and 287 (54.2%) male examinees, while the permanent teeth sample 242 (49.1%) female and 251 (50.9%) male examinees. There were overall 213 (87.7%) female and 259 (90.2%) male examinees with decayed missing filled teeth-dmft 1≤ and 147 (60.7%) and 152 (60.6%) with Decayed Missing Filled Teeth—DMFT 1≤.

Table 2 depicts individual mean dmft/DMFT and SiC index according to the study groups that were analyzed. It also displays a comparison of mean dmft/DMFT and SiC index among different age groups, where all of the comparisons were found to be statistically significant (<0.001) using the One-Way ANOVA test (Table 2).

Table 3 presents the mean numbers of untreated decayed d/D teeth and mean numbers of treated-missing and filled mf/MF teeth among different study groups. A significant difference (<0.00001) was found regarding the average number of active carious lesions and the average number of treated primary and permanent teeth concerning different age groups within the same sex (Table 3) and in the total sample.

After analyzing the data that was obtained related to the mean dmft/DMFT index values, we found a statistical significance (<0.00001) between the sexes. The overall mean dmft was found to be higher in male participants (5.4 vs. 5.1) and mean dt (4.8 vs. 4.1). On the opposite, a higher overall mean DMFT was established in female participants (2.7 vs. 3.0) (Table 4). Based on the comparative analysis of the SiC index (Table 4), we obtain no statistically significant differences regarding different sex.

**Figure 1:**
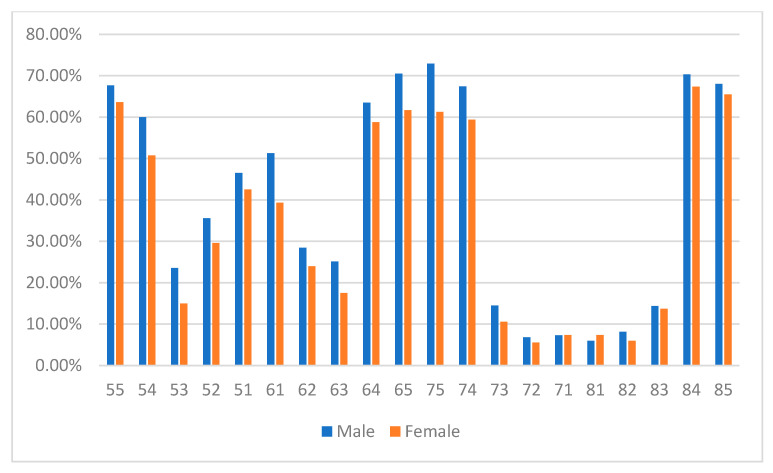
Distribution of dmft according to the tooth type.

**Figure 2:**
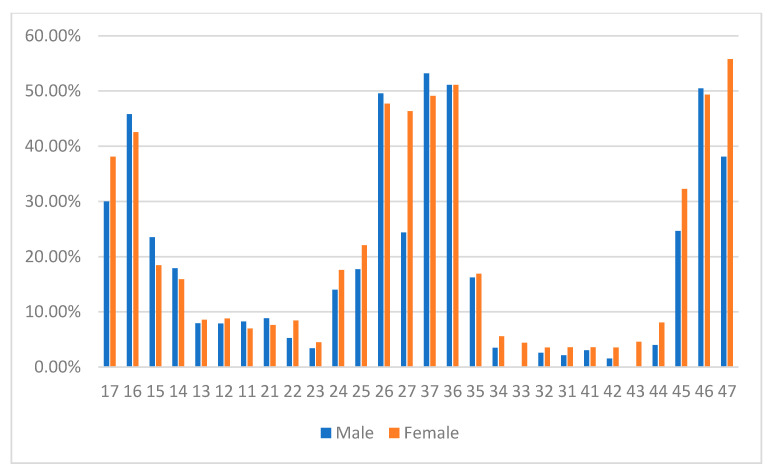
Distribution of DMFT according to the tooth type.

Using the normalized absolute value of the proportional representation of the number of dmft (Figure 1) and DMFT (Figure 2) in relation to the number of teeth that were examined for each type of tooth individually and separately by sex, we observed that in males teeth number 26 (49.6%, *p* = 0.0407), 36 (51.1%, *p* = 0.0337), 46 (50.5%, *p* = 0.365), and 37 (53.2%, *p* = 0.0258) were significantly more affected, whereas in females teeth number 36 (51.1%, *p* = 0.0516) and 47 (51.1%, *p* = 0.0516) were significantly affected. As regards primary teeth, in both sexes, no significant difference was observed in relation to individual teeth.

## 4. Discussion

The results of our research show a very high prevalence of caries in primary and permanent dentition in the sample that we examined. Overall, the prevalence of caries in primary teeth was 89.1%, whereas in permanent dentition, it was 60.7%.

Individual caries indices were also high. The mean DMFT index at the age of ≤5 years was 6.6, whereas the SiC index was 12.2. In the 12–15-year-old age group, the mean DMFT was 6.8, and the SiC was 13.4. The restoration rate was very low in the study groups and, particularly in the age of ≤5 years, where the mean number of treated-missing and filled mf/MF teeth was 0.7, and in the 12–15-year-old age group, where it was 2.4.

In 1981 the World Health Organization—WHO declared that the global goal for oral health by the year 2000 regarding DMFT index for 12-year-olds should not exceed 3 [20]. Subsequently, the WHO set forth new recommendations entitled Oral Health Guidance for 2020 for countries to use when setting their goals and objectives for improving the oral health of their citizens [21]. In accordance with such proposals, Spain defined its basic goals for oral health for the period from 2015–2020 with a goal of achieving a mean DMFT in adolescents aged 12 of ≤1.0, a restoration index of ≥60% for 12 year olds and ≥ 65% for those aged 15; a Significant Caries Index (Sic) target of ≤3 for 12 year olds was set by Spain, and the prevalence of the caries-free population should be at ≥68% and at ≥ 57% for 12- and 15-year olds, respectively [14].

The values of the indices that we found still fall far short of the goals and objectives set out by the WHO. We can also see that the situation has unfortunately not changed compared to the previously published works by researchers from Bosnia and Herzegovina [4,5,6]. Studies from developed countries show a significantly lower prevalence of caries [22,23], especially in permanent dentition [24], while a high prevalence of caries remains evident in deciduous dentition and in underdeveloped countries, populations of lower socioeconomic status, and in minority and immigrant populations [17,25,26,27].

The results of our research demonstrate that in primary dentition, males have a higher overall mean dmft than females (5.4 vs. 5.1). It was also evidently based upon our study that the males we analyzed exhibited a significantly higher Mean number of untreated decayed primary teeth by comparison with the females (4.8 vs. 4.1). By contrast, the females up to age 15 had more DMF teeth (3.0) compared to their male peers (2.7).

A recent study by Shirahmadi et al. found very similar results in children aged 7–12, where males had a mean dmft of 4.9 and females had a mean dmft of 4.5, a difference that was statistically significant. Furthermore, males had a higher percentage of untreated carious lesions compared to females (83.1% vs. 73.2%), which was also statistically significant. In permanent dentition, they identified values of Mean DMFT to be higher in females (2.0) compared to males (1.6) [27].

Furthermore, a study conducted by Prabakar et al. in India, involving children aged 5 to 12 years old, identified a higher overall mean decay, filled teeth—dft, and SiC index in boys (1.4 ± 2.21 and 3.9 ± 2.2) compared to their findings in girls of the same age (1.2 ± 2.1 and 3.5 ± 2.2); however, in permanent dentition, they found no such differences in regard to the gender of the respondents [13]. In their discussion, the authors of the Prabakar study expressed the opinion that, in India, priority is given to boys, and they opined that boys enjoy a more indulgent diet than girls, which is why, in the opinion of the authors, they also tend to have a higher caries prevalence [13]. Despite the existence of various inequalities in some aspects of life in Bosnia, such unequal treatment of children based on gender does not exist in our region. Therefore, a higher incidence of caries in boys in our region cannot be likely attributed to their diet.

Many studies have confirmed that a child’s age is significantly associated with the development of caries [13,25,28]. The results of examined indices were all statistically significant (mean dmft/DMFT, SiC), and moreover, high values of the examined indices were found in all ages. They were particularly high in the ≤5 years old, where DMFT was found to be 7.4 for males and 6.5 for females, which are especially high values for the SiC index in primary teeth (12.2 for males and 12.1 for females). High values were also found in the examined indices for the 12–15 years group for the DMFT and Sic index, where the values that we identified were 4.5 and 12.4 for males, and 6.6 and 14.3 for females. Furthermore, if we consider the ratio of the index for the presence of active carious lesions (dt/DT) and treated teeth mft/MFT, unfortunately, we can determine that there are more untreated carious lesions.

Notably, all of the above-mentioned indexes are higher compared to studies recently published by other authors [13,16,25,27,28]. Many factors could explain such poor dental health in our children; however, one of the most significant factors, in our opinion, is the absence of well-organized, consistently implemented preventive care activities by professional organizations and individuals in B&H.

During the course of our research, we conducted an analysis of the proportional representation of the number of DMFT/dmft in relation to the number of examined teeth for each type of tooth individually, both in males and females. As one may expect, the first and second permanent molars were affected in a higher percentage by comparison with the other types of teeth. It was also found that when compared to other teeth, teeth 26, 36, 46, and 37 were significantly more affected in males, and in teeth 36 and 47 in females.

Notably, research conducted in China, in Zhejiang province, on children aged 6 to 8 years old found that the prevalence of caries in the first permanent molars was significantly higher in girls, and the authors of that study found such prevalence to be associated with the fact that the girls were living in rural areas [29]. Interestingly, this study also identified a higher percentage of caries occurrence on the mandibular molars #36 and #46 in both sexes and in this respect, the results of the Zhejiang study are somewhat similar to our findings in Bosnia.

Additionally, research conducted in São Tomé and Príncipe, in Central Africa, on children aged 11–14 years of age identified a high prevalence of caries on the first permanent molars (68.7%); however, no significant differences in relation to sex were identified, nor were there significant differences in relation to other variables investigated during the course of that study such as age, place of residence, and the number of the children in the family [30].

In the primary dentition, in the sample that we examined, caries was mostly present on the first and second deciduous molars. High values were also found on the maxillary central incisors in both sexes, with tooth 61 being particularly at risk. In fact, 51.3% of these teeth were found to be diseased. Notably, no statistically significant differences were found in deciduous dentition into tooth type, both in males and females.

Contrary to our findings, research by Nomura et al. and Kim et al. found the highest prevalence of caries in the maxillary central incisors [31,32]. In a study conducted in China among children aged 3–4 years of age, Wu et al. also found a higher prevalence of caries in maxillary central incisors in relation to deciduous first and second permanent molars; however, by the age of 5, this finding appears to change, and significantly more caries occurs on the deciduous molars [33]. Such findings should not be surprising, in light of the sequence of tooth eruption and the child’s diet, and the role of certain habits such as using a bottle or eating night meals, whereas, at a later age, the molars are more affected by caries due to their morphological characteristics.

A study conducted by Sirvastava on children aged 3–6 years of age in India showed that the prevalence of caries in primary molars was higher in boys (56.7%) compared to girls (43.3%). The same study found a higher prevalence of caries in mandibular molars compared to the maxillary ones, which was not the case in our study, where we did not find any statistical significance when comparing the prevalence of caries in the mandibular and maxillary molars [34].

The limitations of our study, as with any effort at retrospective research, lies in the fact that the data we used had already been collected, and so certain variables that may have a potential impact on the outcome may have been missing. Additionally, the examinees of this research come from a region around one city, which may imply that the results of our study cannot be extrapolated to the entire country’s population, even though the city of Banja Luka is the largest regional center in the RS government entity and the children are from different socio-economic backgrounds and different schools and preschools from the municipality of Banja Luka. We believe that detecting a possible risk group among such children (in relation to different sex or ages) could prioritize appropriate preventive and therapeutic measures that may be used in other regions of B&H or similar communities with high caries prevalence.

## 5. Conclusions

Taking into account the results that we found during the course of our study, we can see a high caries prevalence and index values in all of the examined groups. In primary dentition, males had a higher overall mean dmftcompared to the females that we studied. We also found a significantly higher Mean number of untreated decayed primary teeth. Females up to the age of 15 were shown to have more DMF teeth compared to their male peers.

The high caries burden found in our research requires a more serious approach to caries treatment in our country and the organization of a comprehensive dental preventive program.

## Figures and Tables

**Table 1:** Distribution of the examinees according to the study groups.

	Number (%) of Examinees with dmft/DMFT 1≤			Number (%) of Caries-Free Examinees			Number (%) of Examinees		
	Male	Female	Total	Male	Female	Total	Male	Female	Total
Primary teeth									
≤5 years old	69 (92.0)	52 (86.7)	121 (89.6)	6 (8.0)	8 (13.3)	14 (10.4)	75 (55.6)	60 (44.4)	135 (25.5)
6–8 years old	85 (90.4)	88 (86.3)	173 (88.3)	9 (9.7)	14 (13.7)	23 (11.7)	94 (48.0)	102 (52.0)	196 (37.0)
9–11 years old	96 (89.7)	58 (89.2)	154 (89.5)	11 (10.3)	7 (10.8)	18 (10.5)	107 (62.2)	65 (37.8)	172 (32.5)
12–15 years old	9 (81.8)	15 (93.8)	24 (88.9)	2 (18.2)	1 (6.3)	3 (11.1)	11 (40.7)	16 (59.3)	27 (5.1)
Total	259 (90.2)	213 (87.7)	472 (89.1)	28 (9.8)	30 (12.4)	58 (10.9)	287 (54.2)	243 (45.9)	530 (100.0)
Permanent teeth									
≤5 years old	2 (15.4)	2 (15.4)	4 (15.4)	11 (84.6)	11 (84.6)	22 (84.6)	13 (50.0)	13 (50.0)	26 (5.3)
6–8 years old	28 (33.3)	35 (39.3)	63 (36.4)	56 (66.7)	54 (60.7)	110 (63.6)	84 (48.6)	89 (51.5)	173 (35.1)
9–11 years old	81 (76.4)	57 (71.3)	138 (74.2)	25 (23.6)	23 (28.8)	48 (25.8)	106 (57.0)	80 (43.0)	186 (37.7)
12–15 years old	41 (85.4)	53 (88.3)	94 (87.0)	7 (14.6)	7 (11.7)	14 (13.0)	48 (44.4)	60 (55.6)	108 (21.9)
Total	152 (60.6)	147 (60.74)	299 (60.7)	99 (39.4)	95 (39.3)	194 (39.4)	251 (50.9)	242 (49.1)	493 (100.0)

Decayed Missing Filled Teeth/decayed missing filled teeth-DMFT/dmft.

**Table 2:** Distribution of mean dmft/DMFT and SiC index among different study groups.

	Male	Female	Total
Mean dmft (sd)			
≤5 years old	7.4 (4.4)	6.5 (4.9)	6.6 (4.6)
6–8 years old	6.1 (4.1)	5.3 (3.2)	5.7 (3.7)
9–11 years old	5.5 (3.6)	4.0 (2.7)	3.8 (3.3)
12–15 years old	2.7 (1.8)	3.3 (2.7)	2.9 (2.4)
*p*	<0.00001 *	<0.00001 *	<0.00001 *
Mean DMFT (sd)			
≤5 years old	0.3 (0.6)	0.3 (0.8)	0.3 (0.7)
6–8 years old	1.1 (1.9)	0.9 (1.4)	1.0 (1.7)
9–11 years old	2.8 (2.3)	2.4 (2.2)	2.6 (2.3)
12–15 years old	6.0 (5.5)	7.4 (6.2)	6.8 (5.9)
*p*	<0.00001 *	<0.00001 *	<0.00001 *
Primary SiC (sd)			
≤5 years old	12.2 (2.9)	12.1(3.0)	12.2 (2.9)
6–8 years old	10.2 (2.6)	9.2 (2.5)	9.7 (2.6)
9–11 years old	9.4 (2.2)	7.1 (1.2)	8.5 (2.2)
12–15 years old	4.5 (1.0)	6.6 (2.4)	5.7 (2.1)
*p*	<0.00001 *	<0.00001 *	<0.00001 *
Permanent SiC (sd)			
≤5 years old	0.8 (1.0)	1.0 (1.2)	0.9 (1.0)
6–8 years old	3.4 (1.7)	2.6 (1.1)	2.6 (1.5)
9–11 years old	5.2 (1.9)	5.0 (2.1)	5.1 (1.7)
12–15 years old	12.4 (4.4)	14.3 (5.4)	13.4 (5.0)
*p*	<0.00001 *	<0.00001 *	<0.00001 *

* One-Way ANOVA; The result is significant at *p* < 0.05. sd-standard deviation.

**Table 3:** Distribution of mean dt/DT and mft/MFT among different study groups.

	The Mean Number of Untreated Decayed d/D Teeth (sd)			The Mean Number of Treated-Missing and Filled mf/MF Teeth (sd)		
	Male	Female	Total	Male	Female	Total
Primary teeth						
≤5 years old	6.7 (4.5)	5.7 (4.7)	6.2 (4.6)	0.6 (0.9)	0.8 (1.1)	0.7 (1.0)
6–8 years old	4.6 (3.8)	4.3 (3.2)	4.4 (3.6)	1.4 (1.4)	1.1 (1.8)	1.2 (1.3)
9–11 years old	4.0 (3.4)	2.9 (2.2)	3.6 (3.2)	1.5 (1.4)	1.2 (1.2)	1.3 (1.3)
12–15 years old	1.8 (1.5)	2.6 (2.6)	2.3 (2.3)	0.3 (0.4)	0.7 (1.0)	0.5 (0.7)
*p*	<0.00001 *	<0.00001 *	<0.00001 *	<0.00001 *	<0.00001 *	<0.00001 *
Permanent teeth						
≤5 years old	0.3 (0.6)	0.2 (0.6)	0.2 (0.6)	0	0.2 (0.6)	0.1 (0.3)
6–8 years old	0.8 (1.5)	0.7 (1.2)	0.8 (1.3)	0.3 (0.7)	0.2 (0.5)	0.3 (0.6)
9–11 years old	1.8 (2.3)	1.4 (1.7)	1.6 (2.1)	1.0 (1.1)	0.9 (0.2)	1.0 (1.1)
12–15 years old	3.7 (4.5)	5.0 (5.4)	4.4 (5.1)	2.4 (2.0)	2.4 (2.4)	2.4 (2.2)
*p*	<0.00001 *	<0.00001 *	<0.00001 *	<0.00001*	<0.00001 *	<0.00001 *

* One-Way ANOVA; The result is significant at *p* < 0.05, sd-standard deviation.

**Table 4:** Comparison of overall mean dmft/DMFT and SiC index.

Caries Index	Male	Female	*p*
Mean dmft (sd)	5.4 (3.9)	5.1 (3.9)	<0.00001 ^1,^*
Mean DMFT (sd)	2.7 (3.5)	3.0 (4.3)	<0.00001 ^1,^*
Mean dt (sd)	4.8 (4.0)	4.1 (3.7)	<0.00001 ^1,^*
Mean DT (sd)	1.7 (2.8)	2.0 (3.4)	0.13362 ^1^
Mean mft (sd)	1.2 (1.3)	1.0 (1.2)	0.11184 ^1^
Mean MFT (sd)	1.0 (1.2)	1.0 (1.5)	0.5157 ^1^
Primary SiC (sd)	10.2 (3.0)	9.2 (3.0)	0.880972 ^2^
Permanent SiC (sd)	5.7 (4.2)	6.2 (5.6)	0.942752 ^2^

^1^ Two-tailed Z test; ^2^ Independent ’*t*’ test; * The result is significant at *p* < 0.05, sd-standard deviation.

## Data Availability

Data will be available upon request to the corresponding author.
